# Organization of primary care and early MOUD discontinuation

**DOI:** 10.1186/s13722-024-00527-w

**Published:** 2024-12-19

**Authors:** Rebecca Arden Harris, Matthew Kearney, Shimrit Keddem, Tara Calderbank, Liza Tomczuk, Justin Clapp, Jeanmarie Perrone, Henry R. Kranzler, Judith A. Long, David S. Mandell

**Affiliations:** 1 Department of Family Medicine and Community Health, Perelman School of Medicine, University of Pennsylvania, Philadelphia, PA USA; 2 Leonard Davis Institute of Health Economics, University of Pennsylvania, Philadelphia, PA USA; 3 Department of Veterans Affairs (VA) Center for Health Equity Research & Promotion (CHERP), Corporal Michael J. Crescenz VA Medical Center, Philadelphia, PA USA; 4 Penn Center for Mental Health, Perelman School of Medicine, University of Pennsylvania, Philadelphia, PA USA; 5 Department of Anesthesiology and Critical Care, Perelman School of Medicine, University of Pennsylvania, Philadelphia, PA USA; 6 Department of Medical Ethics and Health Policy, Perelman School of Medicine, University of Pennsylvania, Philadelphia, PA USA; 7 Dept of Emergency Medicine, Perelman School of Medicine, University of Pennsylvania, Philadelphia, PA USA; 8 Center for Addiction Medicine and Policy, University of Pennsylvania, Philadelphia, PA USA; 9 Department of Psychiatry, Perelman School of Medicine, University of Pennsylvania, Philadelphia, PA USA; 10 VISN 4 MIRECC, Corporal Michael J. Crescenz VA Medical Center, Philadelphia, PA USA; 11 Division of General Internal Medicine, Perelman School of Medicine, University of Pennsylvania, Philadelphia, PA USA

**Keywords:** Opioid used disorder (OUD), Medications for opioid use disorder (MOUD), Buprenorphine, Primary care, Early discontinuation, Clinic operations

## Abstract

- Primary care clinic structures and operations may influence early MOUD discontinuation.
- Flexible scheduling can improve early MOUD retention but must be balanced with clinic efficiency.
- Multidisciplinary teams can improve retention but require additional resources.
- Addressing comorbid pain and polydrug use early in the treatment process can help prevent MOUD discontinuation.

**Supplementary Information:**

The online version contains supplementary material available at 10.1186/s13722-024-00527-w.

## Introduction

More than 5 million individuals in the United States currently live with opioid use disorder (OUD) [1]. In response to the growing need for treatment, primary care physicians (PCPs) increasingly prescribe buprenorphine, [2, 3] a first-line FDA-approved medication for OUD (MOUD) [4, 5]. Primary care offers advantages as a treatment setting for MOUD, including a more private and less stigmatizing environment compared to specialty substance use treatment clinics [6–8]. However, retention rates vary across clinics, with some studies showing up to 50% of patients discontinuing treatment within the first year [9–14]. Similar rates have been observed in both primary care and specialty clinics [15]. Discontinuation of MOUD treatment is associated with increased risks of illicit drug use, morbidity, overdose mortality, legal system involvement, and HIV/HCV transmission [16–20].

About half of patients who discontinue treatment in the first six months do so within 30 days of initiation, although studies differ in their measurement of retention [21, 22]. Patients may be particularly vulnerable to discontinuation during the first four weeks (“early phase”) of MOUD treatment because they have acute medical and psychosocial needs [23] and have not yet formed trusting and supportive relationships with care team members [24, 25].

Although some patient-level factors may influence early MOUD discontinuation [9, 26–31], clinic-level factors, such as care structure and workflow, may also play a role. Examining these potentially modifiable factors could reveal opportunities to improve care delivery, especially for patients who need immediate help.

In this qualitative study, we explored the perspectives of patients, PCPs, and administrators on how the work structures of primary care clinics may contribute to early MOUD discontinuation. We sought to understand how factors such as appointment scheduling, PCP time allocation, and coordination of multidisciplinary services could influence patients' retention or discontinuation of MOUD during the first month of treatment. Additionally, we aimed to capture patients' experiences receiving MOUD in primary care, focusing on aspects that facilitated or hindered their ability to continue treatment while also providing space for patients to discuss other reasons for discontinuation. To gain insights from different vantage points, we interviewed administrators about clinic operations, resources, and priorities, and PCPs about the pressures and constraints they experience in providing MOUD treatment services.

## Material and methods

### Study design and sample

We conducted semi-structured interviews with patients, PCPs, and senior clinic administrators. Eligible patient participants were 18 years or older and had received MOUD from a PCP affiliated with a major mid-Atlantic academic health system with a large catchment area. To capture diverse experiences, patients were selected from the electronic health record using purposive sampling based on race, sex, zip code, and history of early dropout from MOUD treatment, aiming for a balance between those who had and had not dropped out early. We defined early dropout as discontinuation within the first month of MOUD treatment initiation. (We use the terms “dropout” and “discontinuation” interchangeably). To identify potential participants, we utilized electronic medical records to find individuals who started MOUD treatment, distinguishing between patients who received buprenorphine refills after the first month from those who did not. Contacted patients were informed of the study's aims and methods and invited for an interview. Those who consented received a $50 gift card for their time.

The participating clinics, all part of large academic health systems, were generally well-resourced but varied in size and structure for MOUD treatment. Clinic sizes ranged from small (1–2 PCPs who provided MOUD) to large (6 PCPs). Some clinics used a "concentrated arrangement," dedicating specific half-days to OUD care. These sessions often supported by multidisciplinary teams, including social workers and peer recovery specialists. Other clinics adopted a "dispersed arrangement," integrating MOUD into regular primary care visits.

All PCPs interviewed had addiction medicine training, ranging from X-waiver buprenorphine prescribing courses to board-certification in addiction medicine. Both PCPs and administrators volunteered their involvement without compensation.

### Ethics approval

The University of Pennsylvania’s IRB approved this research.

### Data collection

We conducted the interviews by telephone, videoconference, and in-person, depending on participant preference. Interview length ranged from 45–90 min. RAH, MK, JC, and DSM designed the interview guides. Development of content domains and questions was informed by literature review, our previous research, and clinical experience. The guides were organized into three sections: clinic structure and operations, patient care experience, and the potential impact of clinic work structure on early MOUD discontinuation. While some core questions were asked of all participants, guides were tailored to each stakeholder group (see Supplement). For example, patients were asked directly about their reasons for continuing or discontinuing MOUD, while administrators were asked about clinic priorities and operations. PCPs were asked about their experiences providing MOUD treatment, the problems they encountered, and their views on how clinic structures and processes affected early MOUD dropout.

Interviews were audio-recorded and transcribed verbatim or documented through detailed notes taken during the interview. All interviews were de-identified, labeled with only a study ID, and entered in NVivo 1.7.1 (QSR, Doncaster, Australia) for data management and analysis. Interviews were conducted until thematic saturation was reached, defined as the point at which no new themes emerged during interviews and determined by consensus among study investigators.

### Analysis

Analysis followed a modified grounded theory approach [32, 33], meaning that our analysis was largely inductive but was modified to incorporate previously defined coding concepts relating to primary care organization and patient treatment engagement [34, 35]. Two members of the research team (RAH, MK) reviewed the initial transcripts and identified major concepts related to clinic organization and early MOUD discontinuation. The investigators refined the coding structure by merging or removing codes until reaching consensus that no new categories were present. Twenty percent of the transcripts were double-coded with strong agreement among coders (*κ* = 0.88) [36], RAH coded the remaining transcripts, collaborating with two team members (TC, MK) to ensure coding consistency. Discrepancies were resolved through discussion and consensus.

Next, RAH created analytic memos for each code, describing key themes and compiling a list of interviewee quotations. Applying the constant comparison method, the memos were analyzed for consistency and variances, by comparing newly collected data against previously identified categories [33]. The analysis also explored thematic variation among the different participant types (patients, PCPs, administrators), selecting representative quotes for illustration. To maintain anonymity, patient quotes were labeled with the letter "P" followed by a unique numerical identifier, clinician quotes were labeled "C", and administrator quotes were labeled "A".

## Results

### Sample characteristics

We interviewed 30 participants (12 patients, 12 PCPs, and 6 administrators). The patient cohort consisted of 8 males. Four of the patients identified as Black, 1 as being of another race, and 7 as White. Two individuals also identified as Hispanic/Latinx. The patients’ median age was 35 years (IQR: 29–44 years). Using the National Center for Health Statistics' urban–rural classification scheme for counties, half of the individuals lived in large central metro counties, 2 in large fringe metro counties, and 4 in medium metro counties [37].

Eight of the 12 patients had current or previous use of prescription opioids, 9 had used heroin, 9 fentanyl, 9 benzodiazepines, 6 cocaine, and 5 methamphetamines. Four patients experienced 1 or 2 overdose events and 3 experienced 3 or more (Table 1). None of the patients were treatment naïve, and half experienced early MOUD dropout.

**Table 1:** Characteristics of patients (n = 12)

	N (%)
*Age*	
18–24	1 (8%)
25–44	8 (67%)
45–64	3 (25%)
*Gender*	
Male	8 (67%)
Female	4 (33%)
*Race*	
Black	4 (33%)
White	7 (58%)
Other	1 (8%)
*Ethnicity*	
Hispanic/Latinx	2 (17%)
Non-Hispanic/Latinx	10 (83%)
*NCHS urban–rural classification*	
Large central metro	6 (50%)
Large fringe metro	2 (17%)
Medium metro	4 (33%)
*Structural factors associated with early discontinuation*^a^* (n = 11)*	
Unemployed	5 (45%)
In past 3 months,	
Experienced food insecurity	3 (27%)
Lived in unstable housing	1 (9%)
Experienced transportation barriers	3 (27%)
Experienced new legal issues	3 (27%)
In next 3 months,	
Worry that housing will become unstable	5 (45%)
*Substance use history*	
Benzodiazepines	9 (75%)
Cocaine	6 (50%)
Fentanyl	9 (75%)
Heroin	9 (75%)
Methamphetamine	5 (42%)
Prescription opioids	8 (67%)
*Co-morbidity*	
Chronic pain	7 (58%)
*Number of drug overdose events*^a^* (n = 10)*	
0	3 (30%)
1–2	4 (40%)
3 or more	3 (30%)
*Number of early treatment discontinuations*	
0	6 (50%)
1–2	5 (42%)
3 or more	1 (8%)

^a^Ns refer to number of participants who responded to the question

The clinician group consisted of 12 PCPs; 5 male, 1 Black, 2 Asian, and 9 White individuals. Of the 6 clinic administrators, 3 were male, 1 non-White, and 5 White (Table 2).

**Table 2:** Characteristics of PCPs (n = 12) and clinic administrators (n = 6)

	N (%)
*PCPs*	
Gender	
Male	5 (42%)
Female	8 (58%)
Race/ethnicity	
Asian	2 (17%)
Black	1 (8%)
White	9 (75%)
*Administrators*	
Gender	
Male	3 (50%)
Female	3 (50%)
Race/ethnicity	
Non-White	1 (17%)
White	5 (83%)

### Themes

**Fig. 1:**
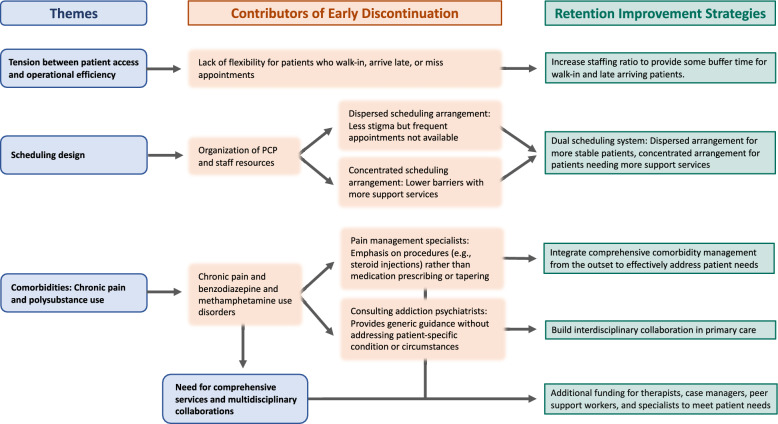
Contributors to early MOUD discontinuation and retention strategies

Four main themes emerged from the interviews, each related to early treatment challenges: the importance of balancing patient access and operational efficiency, the impact of different care delivery and scheduling approaches, the need to address comorbidities, and the value of effective collaboration across disciplines (Fig. 1, Table S1).

#### Theme 1: balancing patient access and operational efficiency

Clinics aimed to increase appointment availability to prevent early MOUD discontinuation while maintaining smooth patient flow. While many patients attended scheduled appointments without issue, some individuals, particularly those with severe OUD and those juggling work or other commitments, reported difficulties that led to early MOUD discontinuation. Severe OUD was defined through a comprehensive assessment of participants' substance use patterns and their effects on daily life, including the frequency and intensity of opioid use, polysubstance use, history of overdoses, and overall functioning. One patient (P09), who discontinued early to self-manage OUD, recalled the lack of access at their primary care clinic as the reason for dropout: "There were only certain doctors [who prescribed MOUD] and they were only there once, maybe twice a week."

PCPs and administrators recognized the complications that offering walk-in appointments could create for planning and staffing. PCP C05 mentioned their clinic’s decision to discontinue walk-in hours:

“We shut down the walk-in clinic. PCPs assigned to it hated it because they had anywhere between 2 and 25 patients, so much grumbling and chaos, full waiting room. When someone walks in now, we try to put the patient on the schedule, refill their MOUD, and book a close follow-up appointment. But generally, we try to discourage walk-ins.”

Administrator A03 also explained that there are potential financial losses for primary care walk-in clinics with consistently low patient show rates: “Anytime there is a mismatch in what you’re paying support staff and PCPs and utilization of team, it’s going to threaten your finances.”

Accommodating walk-in and late-arriving patients to a PCP’s schedule sometimes made it difficult for PCPs to stay on time, maintain quality care, and avoid burnout. Administrator A06 suggested that effective triage may help manage these problems:

“If a patient is late, it throws off the care delivery for every other patient. Walk-in patients come in without prior triage, leading to situations where they may be too sick for primary care or not sick enough to warrant immediate attention.”

Clinics sought to strike a balance between offering flexible scheduling to prevent MOUD discontinuation and ensuring efficient care delivery for patients who arrived on time. Another administrator (A04) spoke to the importance of serving different patient populations:

“We’re dealing with different patient populations. What one group appreciates, another may find inconvenient or frustrating. A more flexible system greatly benefits some people, but it also leads to longer wait times for those who show up on time.”

#### Theme 2: care delivery and scheduling

We identified two distinct systems for scheduling OUD patients with PCPs: the "dispersed arrangement" and the "concentrated arrangement." Each approach has its strengths and weaknesses.

In the dispersed arrangement, OUD patients were integrated into the primary care caseload and received MOUD from their assigned PCP, which may help reduce stigma. For these reasons, PCP C10 preferred the dispersed arrangement:

"In my practice, I see about 20–25 patients daily and about three of them require Suboxone; it's a healthy mix. It reduces stigma and normalizes the treatment. When I'm away from the office, other providers prescribe refills the way we do for all care."

Patients in the dispersed arrangement sometimes faced difficulties scheduling urgent appointments with their assigned MOUD provider, as PCPs' schedules were booked weeks or even months in advance. To accommodate those needing prompt care, exceptions were usually made to standard scheduling procedures. A patient liaison, such as a case manager or peer recovery specialist, or the patients themselves would directly contact the PCP to arrange for the patient to be double-booked or to receive a bridging MOUD prescription until a clinic visit could be scheduled. This ad-hoc process allowed for flexibility. Patient P04 had a positive experience:

“If I call her, [the doctor] stops everything she was doing, if she’s in the hospital, she’s going to call me. Because I don’t call unless it’s like an emergency. So, if I call or if I text, she’s on it. If I say I can’t [make the appointment], I have that trust with her that she’ll give me a refill for the four weeks.”

Although these measures were an important interim solution to ensure patients received necessary care, they raised concerns about resource constraints, PCP burnout, and inconsistent application across providers, resulting in uneven access for some patients. This was the case for patient P05:

"Well, it used to be easy, it's not easy anymore. There used to be a care coordinator. I would just text her and she would relay the message and that they would get back to me super quick. But the doctor I have now, it's like two days before I hear back so if there's any issues, I'm really not in good condition."

The concentrated arrangement designated specific half-days for OUD care, usually once or twice a week. These "specialty subclinics" were staffed by MOUD-experienced PCPs and often supported by a team that included part-time case managers, pharmacists, and peer support workers. Administrator A03 pointed out the benefits of this approach:

“I think the concentrated approach is better. Patients are more likely to get a consistent level of knowledge and expertise. There’s a dedicated support staff who knows the patients and the drill. I think it’s easier to follow-up with patients and make sure they get in. Regular primary care clinic can be difficult to get in. The patient has to call the call center, wait on the phone…they may be offered an appointment that is weeks off. This way, you are offering concierge care within a large, primary care practice. This is especially important for patients vulnerable to early dropout.”

Patient P12 attested to the positive impact of the dedicated support staff in this model:

“I have a care coordinator or care manager, she’s awesome. She’s always there [on MOUD clinic days], she calls me all the time, she’s always checking on me. It’s like a second mom.”

The concentrated arrangement was partially designed to accommodate late-arriving patients and provide same-day telemedicine alternatives for those who might otherwise miss their appointments, providing timely and flexible access to care. Administrator A06 elaborated:

“With the concentrated approach, we can have a different check-in process. Because patients show up late, we suspended our usual late policy. We can cloister the patients in that way, they get a little less push back if they don’t follow the typical rules.”

The concentrated arrangement's restricted schedule could pose barriers for some patients, particularly in clinics offering MOUD care only once or twice a week. To address urgent needs between appointments, clinics sometimes implemented special procedures outside of standard operations, facing limitations similar to those in the dispersed arrangement.

Both arrangements also presented obstacles to maintaining PCP continuity, as PCPs divided their time between primary care and other academic or clinical roles, which made it difficult to sustain consistent patient relationships. Administrator A04 questioned the feasibility of maintaining PCP continuity for patients with OUD while simultaneously managing other obligations:

"I’m uncertain if it’s feasible to align part-time providers in such a way that prioritizes continuity for patients, especially those on MOUD, while also allowing providers to meet their productivity goals, maintain their wellness, know when they will be leaving clinic to go home, and can participate in an incentive program."

Some patients expressed frustration with the lack of continuity, potentially eroding their motivation to return for follow-up appointments. Patient P08 shared:

“I have to explain [my problem to the first doctor]…then the next time I go, instead of being able to follow up with that, I have to re-explain the whole story to the next person because…we're not on the same page that I was with this other person.”

PCP C09 elaborated on the pros and cons of PCP continuity:

"When it comes to continuity of care, there's a mixed benefit there. In the context of perinatal care, where there's a risk of child protective services taking the child away, it's easier for the patient to develop trust with one PCP and not have that trust spread out over all the PCPs. However, this can lead to counter-behavior or splitting behavior, where the patient says, 'I’m only going to get care from one person and refuse to see anyone else.' That's not okay either, as it can limit the patient's access to comprehensive care and put undue pressure on a single PCP. So, broadly speaking, while continuity of care benefits patients in terms of building trust and rapport, it may not always be beneficial for PCPs in terms of workload distribution and team-based care."

#### Theme 3: addressing comorbidities

Participants highlighted the importance of addressing comorbid conditions, such as chronic pain, methamphetamine use disorder, and benzodiazepine use disorder, to prevent early dropout. While MOUD effectively mitigated withdrawal symptoms and reduced opioid cravings, some patients with severe chronic pain required additional support. PCPs prescribed opioid alternatives to avoid exacerbating dependency, but in some cases, these treatments were not sufficient, leading to disengagement and return to use. Patient P06 expressed their concerns:

"Suboxone has been helping me because I haven't been getting sick, but when I'm going through pain, I still need opioid pills to ease it. However, I'm very scared because I don't want my friends to give me something that could cause me to overdose like I did previously."

To address benzodiazepine use disorder, PCPs carefully tapered or limited benzodiazepine prescriptions due to concerns about overdose and diversion [38]. While these measures were implemented with patient safety in mind, for some patients, they may have contributed to seeking illicit alternatives and increased pressure to drop out early. Patient P06 expressed their frustration and describes the self-help measures taken when the clinic did not meet their needs:

“I suffer from anxiety. I suffer from PTSD. I really suffer. I tried explaining if he was giving me a month's worth, I wouldn't have to [take street] Xanax.”

Another patient (P09) attributed their return to drug use to their inadequately addressed benzodiazepine use disorder:

“It was because I was struggling with not only opioid dependence but also with benzodiazepines and [the PCP] could only do so much for that, and I couldn't really get help for that. So, I just kept using that and I ended up using other substances as well.”

The absence of FDA-approved medications for methamphetamine use disorder [39] and extended wait times for specialized behavioral health services resulted in a narrow OUD-focused treatment approach. This may have contributed to early discontinuation for some patients whose co-occurring conditions were not adequately addressed. Patient P09 explained:

“I don't blame them because they don't want to support me abusing drugs or narcotics. But…I have to go undertreated because the medications that they are giving to me don't work very well.”

Several PCPs acknowledged the varying levels of expertise and comfort among the MOUD prescribers in managing complex patients, a sentiment captured by PCP C10:

“A few of us started offering MOUD without formal training in addiction medicine. We knew it was needed and got the X-waiver to prescribe buprenorphine. As some cases are getting more complex, more expertise and support would be helpful.”

PCPs emphasized the importance of collaboration among the healthcare team, especially regarding more complicated patients. PCP C09 observed:

"Collaboration among our primary care-addiction medicine team would help improve outcomes. In theory, we should be able to accomplish this since we have all the necessary folks on the team. In the past, we used to have recovery rounds where periodically we would meet to discuss complicated patients. A system like that could work well if there was someone to coordinate the process and block out dedicated provider time to review cases."

#### Theme 4: collaborating across disciplines

Effective coordination with pain management and mental health specialists may be needed to provide comprehensive care to patients with complex needs and to deter early MOUD dropout. While difficulties in this area were identified, participants also noted potential opportunities for improvement.

PCPs and administrators reported that pain management specialists strongly preferred procedural interventions over opioid prescription management, which was thought to stem from the discipline's focus on reducing misuse and overdose. This cautious stance sometimes led to a gap in cooperation with PCPs in meeting patients' needs. Administrator A05 pointedly observed:

“None of the pain management specialists engage in chronic opioid prescribing. They don't assist with tapers, they don’t step in. Their focus is interventional. They’ve abandoned the space.”

PCPs also encountered obstacles in providing early OUD treatment for patients with more severe mental illness or polysubstance use. This was primarily attributed to the limited availability of psychiatrists and mental health clinicians who are skilled in treating co-occurring disorders. PCP C08 echoed the experiences of the PCPs:

“Scheduling appointments with psychiatry is a persistent problem, possibly related to insurance limitations with Medicaid. We've attempted coordination with the community-based behavioral health programs, but it's been nearly impossible. It can take as long as year to get an appointment.”

To bridge this gap, some PCPs had access to a collaborative care consulting psychiatrist who provided them with guidance in managing complex cases. While the PCPs generally valued having access to this resource, they sometimes found that the broad nature of this advice did not translate well to the specific needs of patients:

“It’s hard to implement the recommendations of a collaborating psychiatrist. There is too much distance between the psychiatrist and the patient. There needs to be a relationship and ability to engage in motivational interviewing because patients don't just go along with recommendations. Otherwise, I feel like I’m getting information that I could get from a textbook, it’s not personalized or very helpful.” C10

Administrators and PCPs more experienced in treating OUD said they were open to adopting standard of care interventions like contingency management for co-occurring stimulant use disorder [40, 41]. Successful implementation would depend on reliable funding, easy integration into current clinic practices, and protocols complying with federal and state laws. PCP C09 summarized:

“This approach [contingency management] could be viable if it's well-managed, with protected time for the team to review the caseload, instead of relying on reading clinic notes. But someone must coordinate it, take ownership, and block PCP time for care coordination activities. And we don't get paid for care coordination.”

## Discussion

The present study explored potential connections between primary care clinic structures and early MOUD discontinuation from the perspectives of patients, PCPs, and administrators. Our findings suggest that clinic-level factors, such as appointment accessibility, care delivery models, and interdisciplinary collaboration, can influence patients' ability to remain engaged in MOUD treatment during the critical early phase. Based on these findings, we propose several modifications to primary care clinic structure, operations, and priorities to reduce early MOUD discontinuation and opioid-related harms.

Participants described the importance of striking a balance between offering flexible scheduling and walk-in appointments on the one hand and maintaining clinic efficiency and preventing provider burnout on the other. While some patients were able to attend appointments regularly, others, particularly those with severe OUD and competing responsibilities, struggled to keep scheduled visits and benefited from the availability of same-day or walk-in appointments. To accommodate diverse needs, clinics might consider incorporating same-day in-person or virtual appointment slots along with buffer periods in PCP schedules [42]. However, the practicality of these strategies may be limited as they require patients to have access to wireless devices and clinics to have flexible PCP and staff time [43].

Our study identified two distinct care delivery models: the "dispersed arrangement," where MOUD is integrated into regular primary care visits, and the "concentrated arrangement," where dedicated clinic sessions are allocated for OUD treatment. While the dispersed model may help reduce stigma, the concentrated model was perceived as providing more specialized care and resources. However, both models presented challenges related to provider continuity and availability, particularly in academic settings where PCPs have multiple obligations. A dual system allowing patients to transition between concentrated and dispersed care based on their needs and clinical stability may be optimal, but further research is needed to assess the feasibility and effectiveness of this approach.

Participants also emphasized the impact of co-occurring conditions, such as chronic pain, mental illness, and polysubstance use, on early MOUD discontinuation. PCPs cited limited access to specialist care and insurance restrictions as difficulties in managing these complex patients. While participants did not provide specific recommendations, their experiences underscored the need for integrated treatment approaches that address patients' varied needs from the start. Some clinics may already utilize team members such as behavioral health therapists, case managers, and peer recovery support workers to varying degrees, but our findings suggest a need for more formalized and integrated roles, particularly in the early stages of MOUD treatment.

Our findings should be interpreted with several limitations in mind. This study focused on clinic-level factors; however, patient-level, socioeconomic, and political factors may also play an important role in influencing MOUD retention. Additionally, the research was conducted in academic primary care clinics in a specific region, so the findings may not generalize to other settings, particularly rural clinics or those with different funding structures or patient populations. The "concentrated arrangement" observed in some of the more resourced academic primary care clinics may not be typical of primary care settings. Similarly, the level of addiction expertise among the PCPs in our study may be higher than what is commonly found in primary care practices.

The possibility of participation bias, where those who agreed to be interviewed may not fully represent the broader population, as well as recall bias, where participants' recollections of past events may be inaccurate or incomplete, should also be acknowledged as potential study limitations.

## Conclusion

This qualitative study offers insights into how clinic-level factors may impact early discontinuation of MOUD. Though patient-level factors almost certainly contribute, our findings suggest that improving appointment access, implementing hybrid care delivery models, and fostering interdisciplinary collaboration could help primary care clinics better support patients in the crucial early phase of MOUD treatment.

## Supplementary Information

Supplementary Material 1. Table S1. Clinic-related barriers and facilitators to early MOUD retention in primary care

## Data Availability

The qualitative data generated in this study are not publicly available to protect participant privacy and confidentiality. However, the data may be available in an anonymized form from the corresponding author upon reasonable request and execution of appropriate Data Use Agreements.

## Abbreviations

MOUD: Medication for opioid use disorder
OUD: Opioid use disorder
PCP: Primary care provider
