# Supplementary material for: Organization of primary care and early MOUD discontinuation
Source: Addict Sci Clin Pract. 2024 Dec 19;19:96. doi: 10.1186/s13722-024-00527-w (PMC11658460; doi:10.1186/s13722-024-00527-w)
Supplement: Supplementary file 1 — Supplementary Material 1. Table S1. Clinic-related barriers and facilitators to early MOUD retention in primary care [file 13722_2024_527_MOESM1_ESM.pdf]

Patient Interview Guide

[After completing informed consent protocol]

I. For all interviewees:

1. Can you tell me about how you chose to go to primary care for help with your opioid use?
    a. How did you connect with primary care for the treatment of opioid use?
    b. Were you looking to start medications for opioid use disorder (MOUD) at primary care?
    c. Were you referred? (e.g., by the Emergency Department, a friend, an outreach or peer worker)
    d. Did your PCP bring up your opioid use and offer MOUD?

2. How long did it take to get your first appointment?

3. How long did it take to get your first buprenorphine prescription?

4. Had you sought treatment for opioid use disorder before?
    a. How was this time different?

5. What had it been like to go to primary care before seeking treatment for opioid use?

6. What were your goals for treatment?
    a. Abstinence (opioid only vs. all)
    b. Reduction in drug use (opioid only vs. all)
    c. Safer drug use, screening for infections, harm reduction supplies
    d. Addressing other medical or mental health concerns
    e. Assistance with attaining social services
    f. Pain management

7. What was it like going to a primary care clinic for treatment for opioid use during that first day of treatment?
    a. Was it what you expected?
    b. (If patient had received MOUD from specialty clinic in the past): How was it different than going to a specialty clinic?
    c. What did you know about the doctor?
    d. What did you know about the clinic?
    e. Did it change for the better or worse during the first few weeks?
    f. Were you referred to the clinic or to a particular doctor?

8. What was induction like?

9. What was it like talking with your PCP?

10. Did you see the same doctor each visit, or did the doctors change at each visit?
    a. What was it like to talk about drug use?
    b. Were you able to raise all of your concerns?
    c. Did you get the help you were looking for?
    d. How do you feel you were treated?

11. Most patients experience some slip-ups. It's very common. Did you have any slip-ups/return to use?

12. Were you using other drugs at the time? Did that change at all?
    a. If so, how did the clinic/PCP respond or support you?

13. How frequently did you have visits during the first month?

14. What were the urine drug testing requirements during the first month?

15. Were there telehealth options during the first month?

16. What other services did they provide?
    a. Were there services you wished they provided, but didn't?
    b. What services did you access outside of the primary care clinic?
    c. How were these services helpful?

17. Would group visits with other patients be helpful? Or group therapy?

18. What do you think of the amount of time with the provider?
    a. More time with PCP or less? Just right?

19. How do you get in touch with your doctor?
    a. Was it easy/hard?

20. Had you tried suboxone before this time?
    a. How was this time different than last time?

21. Had you tried non-prescribed Suboxone before? How did you use it?

22. Have you worked with a peer recovery specialist?
    a. Can you tell me about that?

23. If you switched clinics, why did you prefer the other clinic?

24. What was the vibe of the clinic?

25. Some people tell us that they use different types of substances while taking suboxone. How could the clinic better support people who use multiple substances while in treatment for OUD?

26. Some people have told us that they have felt under pressure to sell their medications because they need money to live. How could the clinic better support patients in this situation?
    a. Have you ever felt those kinds of pressure?

II. For interviewees who were retained at 4 weeks:

1. What things helped you come back to the clinic for your appointments?
    a. Good relationships with clinic staff including PCP
    b. Social support
    c. Patient-friendly clinic services
    d. Appointment reminders
    e. Flexible clinic schedule
    f. Available MOUD options (suboxone, sublocade, naltrexone)
    g. Other:____

2. What were some of the problems you had to face in coming to the clinic?
    a. Competing life activities (child or elder care, work, school)
    b. Feeling sick
    c. Stigma
    d. Depression or mental illness
    e. Expensive or unreliable transportation
    f. Insufficient health insurance
    g. Forgetfulness
    h. Unpleasant experience coming to clinic and/or seeing the PCP
    i. Difficult relationships with clinic staff including PCP
    j. Difficulty scheduling timely appointment
    k. Inconsistent, unstable, or inadequate housing
    l. Other substance use
    m. Justice system involvement
    n. Unexpected crisis
    o. Cost of health care services
    p. Inflexible clinic/administrative procedures or appointment schedule
    q. Lack of MOUD options (methadone)
    r. Hospitalization

III. For interviewees who discontinued MOUD by 4 weeks:

1. What were the reasons you didn't return for care with your PCP?

2. What was the main reason you didn't return for care with your PCP?
    a. I'm going to list the reasons you just mentioned -- please tell me which was the main reason.
    b. Was it something else: ____

3. Did the clinic experience affect your decision to not return to care? How so?

4. What improvements to the clinic experience could have helped you stay in care?
    a. Walk-in hours
    b. Evening hours
    c. Telemedicine
    d. Free harm reduction kits
    e. Transportation assistance
    f. Child and elder care assistance
    g. Peer recovery specialist
    h. Regular peer/mutual-aid group meetings at the clinic
    i. Lunch and Learns
    j. Computer/wi-fi/phone access
    k. Other: ____

5. If you found a different clinic for OUD care, why did you prefer this other clinic?

6. Are you still taking MOUD, either prescribed or otherwise?

7. If you stopped taking it, how are you doing?

4. If you subsequently re-engaged in care, what were the reasons you went back to the PCP after the period you were out of care?

5. Did the clinic experience affect your decision to not return to care initially? How so?

6. How could the clinic experience be improved?

Thank you

Thank you for sharing your insights and experiences. Your perspective is invaluable to our work in improving primary care programs for individuals in the first month of OUD treatment.

PCP Interview Guide

[After completing informed consent protocol]

1. How long have you been practicing at this clinic?

2. How long have you been providing OUD care at this clinic?

3. How many hours per week do you typically spend providing OUD clinical care?

4. How many patients in your current practice (panel) do you know have OUD?

5. In your estimation, what percent of your patients have severe OUD?

6. MOUD care can be provided in different ways: through a 'concentrated model' where specific clinic half-days sessions are reserved to seeing patients with OUD or via a 'dispersed model' where patients with OUD are integrated into the everyday schedules of PCPs.

   From your perspective, which approach is a better fit for an academic primary care clinic? For preventing early dropout?

7. How does your clinic accommodate walk-in and late patients with OUD?

8. How much scheduling flexibility can your clinic build into its operations to accommodate patients with higher no-show, walk-in, and late arrival rates?

9. Primary care practices often use a "group coverage model" where a patient may see a different provider at each visit, but one PCP is responsible for the patient's overall care. In your practice, do you work within a PCP group to provide OUD care for a patient?
   a. If so, how does it work?

10. Some vulnerable patients, such as those starting MOUD, may do better seeing the same PCP at each visit. Would you have any suggestions for how to increase continuity for patients early in OUD treatment stage who may need it the most?

11. What support do you have to help manage patients with more complex needs (i.e., polysubstance use, chronic pain)?

12. When do you order UDT as part of your OUD practice?

13. Does your clinic link patients with OUD to resources (e.g., transportation assistance, medication discount programs, housing support, and insurance and legal resources)?
    a. If so, how?
    b. Is the linkage process effective? Does it produce prompt reliable results?

14. When do you use telemedicine (e.g., virtual clinic appointments, phone calls, secure portal messaging) to provide OUD care?

15. The research literature suggests that patients with OUD, especially those who are also dealing with chronic pain or benzodiazepine use disorder, might benefit from a collaborative treatment model. This approach could involve addiction psychiatry, behavioral health, physical therapy, and pain management specialists working with PCPs to create a comprehensive plan. May I have your thoughts on a collaborative model? Is it feasible in your clinic?

16. My next question is about an experimental treatment approach, Contingency Management (CM), for patients with stimulant use disorder. As you know, CM is a type of psychosocial treatment that rewards patients with small tokens, money, or prizes for staying substance-free. It is one of the few treatments for stimulant use disorder that has shown some effectiveness. Up to now, it's mostly been offered in substance use specialty clinics. What do you think about offering CM in primary care?

17. Your suggestions to make MOUD services more patient-centered?

18. Your suggestions for reducing early MOUD discontinuation?

Thank you
Thanks for taking the time to talk with me. Your observations and views are greatly appreciated!

Administrator Interview Guide

[After completing informed consent protocol]

1. How long have you been in your role as a leader of clinical operations (e.g., medical director, division chief, clinical operations director)?

2. Do any of your clinics have dedicated practice sessions for your OUD patients? For those that do:
    a. How many MOUD clinic sessions per week, and are any of them evening sessions?
    b. How many providers and patients per session?
    c. Are any (some, all) resident precepting sessions?

3. MOUD care can be provided through a 'concentrated model' (specific clinic half-days reserved for OUD patients) or a 'dispersed model' (OUD patients integrated into everyday PCP schedules).
    a. From an administrative perspective, which approach is a better fit for academic primary care clinics like yours, considering the OUD patients' higher no-show, walk-in, and late-arrival rates and Medicaid being the most frequent payer?

4. How do MOUD clinics compare with ordinary continuity care in terms of revenue per session (resident and non-resident clinics)?
    a. Is this a consideration? What other benchmarks do you use?
    b. Is your current payment model for MOUD services sustainable over the long term? How so?

5. Are no-show rates in MOUD clinics compared to non-MOUD clinics something that you measure or consider?

6. How does your clinic address missed MOUD appointments (e.g., double-booking, appointment reminders)?

7. Are there additional non-billable costs for administration time and non-clinical staff in providing MOUD? What are they?
    a. Is the MOUD service volume sufficient to cover these costs?

8. How much scheduling flexibility can a clinic build into its operations to accommodate patients with higher no-show, walk-in, and late arrival rates?
    a. At what point does adding more flexibility seriously impact clinical operations and revenue?

9. Could a policy of squeezing in walk-ins or late arrivals work in a large practice? Would it face resistance from staff and clinicians? What would make it more feasible and acceptable?

10. Many academic clinics are moving to a team-based care model. How can clinics increase continuity for vulnerable patients, such as those starting MOUD, who may benefit from seeing the same PCP at each visit?

11. Discharges due to non-payment, UDT not revealing buprenorphine (suggesting diversion), or consecutive refill requests without an appointment are recurring themes in OUD treatment literature. Is this a problem in your experience? How can clinics address it?

Some advocates are proposing changes to the MOUD clinical model in primary care. I'd like to get your thoughts on the feasibility of these proposals in an academic setting:

- Walk-ins for same day prescription for new and returning patients
- Evening and weekend hours
- Expanding telemedicine to replace in-person visits, including new patient visits
- Provision of harm reduction supplies (naloxone, syringes, fentanyl test strips)

12. The addiction medicine literature suggests that patients with OUD, especially those who are also dealing with chronic pain or benzodiazepine use disorder, might benefit from a collaborative treatment model. This approach could involve addiction psychiatry, behavioral health, physical therapy, and pain management specialists working with PCPs to create a comprehensive plan. May I have your thoughts on the merits and feasibility of a collaborative model? What are the obstacles to implementation?

13. My last question is about an experimental treatment approach, Contingency Management (CM), for patients with stimulant use disorder. As you know, CM is a type of psychosocial treatment that rewards patients with small tokens, money, or prizes for staying substance-free. It is one of the few treatments for stimulant use disorder that has shown some effectiveness. Up to now, it’s mostly been offered in substance use specialty clinics. What do you think about offering CM in primary care? Is it feasible? What are the barriers?

14. Other suggestions for reducing early MOUD treatment dropout in primary care?

Thank you

Thank you for taking the time to talk with me today. Your observations and views are greatly appreciated!

Table S1. Clinic-related barriers and facilitators to early MOUD retention in primary care

Clinic Factors	Barriers	Facilitators
Appointment Accessibility	• Limited availability of MOUD-prescribing PCPs<br>• Rigid scheduling systems<br>• Long wait times for appointments	• Flexible scheduling options<br>• Same-day or walk-in appointments<br>• Telemedicine alternatives
Care Delivery Model	• Lack of PCP continuity<br>• Resource-intensive integrated care team model (e.g., cost of co-located social worker, therapist, peer recovery specialist)	• Concentrated arrangement with co-located ancillary services for patients with higher needs<br>• Dispersed arrangement for more stable patients, reduces stigma<br>• Hybrid model enabling transition between care levels and resource conservation
Addressing Comorbidities	• Limited access to pain management specialists<br>• Long wait times for mental health services<br>• Lack of FDA-approved medications for methamphetamine use disorder	• Collaborative care with consulting psychiatrists<br>• Integration of behavioral health therapists<br>• Implementation of evidence-based interventions (e.g., contingency management)
Interdisciplinary Collaboration	• Siloed care between primary care and specialists<br>• Limited availability of addiction medicine expertise	• Regular team meetings to discuss complex cases<br>• Integrated roles for case managers and peer recovery support workers<br>• Coordinated care between PCPs and specialists
Clinic Operations	• Potential financial losses from low show rates<br>• PCP burnout from overbooked schedules	• Buffer appointment slots in PCP schedules
Patient-centered Care	• Lack of personalized treatment plans<br>• Inadequate attention to patient preferences and needs	• Tailored treatment approaches (e.g., benzodiazepine tapering protocols)<br>• Patient participation in treatment decisions<br>• Addressing social determinants of health
